# An Abundance of Ubiquitously Expressed Genes Revealed by Tissue Transcriptome Sequence Data

**DOI:** 10.1371/journal.pcbi.1000598

**Published:** 2009-12-11

**Authors:** Daniel Ramsköld, Eric T. Wang, Christopher B. Burge, Rickard Sandberg

**Affiliations:** 1 Department of Cell and Molecular Biology, Karolinska Institutet, Stockholm, Sweden; 2 Department of Biology, Massachusetts Institute of Technology, Cambridge, Massachusetts, United States of America; EMBL, Germany

## Abstract

The parts of the genome transcribed by a cell or tissue reflect the biological processes and functions it carries out. We characterized the features of mammalian tissue transcriptomes at the gene level through analysis of RNA deep sequencing (RNA-Seq) data across human and mouse tissues and cell lines. We observed that roughly 8,000 protein-coding genes were ubiquitously expressed, contributing to around 75% of all mRNAs by message copy number in most tissues. These mRNAs encoded proteins that were often intracellular, and tended to be involved in metabolism, transcription, RNA processing or translation. In contrast, genes for secreted or plasma membrane proteins were generally expressed in only a subset of tissues. The distribution of expression levels was broad but fairly continuous: no support was found for the concept of distinct expression classes of genes. Expression estimates that included reads mapping to coding exons only correlated better with qRT-PCR data than estimates which also included 3′ untranslated regions (UTRs). Muscle and liver had the least complex transcriptomes, in that they expressed predominantly ubiquitous genes and a large fraction of the transcripts came from a few highly expressed genes, whereas brain, kidney and testis expressed more complex transcriptomes with the vast majority of genes expressed and relatively small contributions from the most expressed genes. mRNAs expressed in brain had unusually long 3′UTRs, and mean 3′UTR length was higher for genes involved in development, morphogenesis and signal transduction, suggesting added complexity of UTR-based regulation for these genes. Our results support a model in which variable exterior components feed into a large, densely connected core composed of ubiquitously expressed intracellular proteins.

## Author Summary

A variety of genes are active within the nuclei of our cells. Some are needed for the day-to-day maintenance of cell functions, while others have roles that are more specific to certain tissues or particular cell types; for example, only the pancreas produces insulin. As a result, every tissue has its own profile of gene activity. Since active genes produce RNA, tissue differences in gene activity can be probed by characterizing the RNA they contain. Essentially the entire set of RNAs or ‘transcriptome’ has been sequenced from various tissues, and we used these data to compare the degree of specialization of different tissues and to investigate the set of ‘core’ genes active in every tissue. A central observation was that there are an abundance of such core genes, and that these genes account for the majority of the transcriptome in each tissue. These findings will aid in the understanding of what makes tissues, and cell types, different from each other and what each requires to function.

## Introduction

A fundamental question in molecular biology is how cells and tissues differ in gene expression and how those differences specify biological function. A related question is what part of the cellular machinery represents housekeeping functions needed by all cells and how many genes encode such functions. The transcriptomes of mammalian tissues have been extensively studied using methods such as reassociation kinetics (Rot) [1], serial analysis of gene expression (SAGE) [2], microarrays [3],[4], and sequencing of expressed sequence tags (ESTs) and full length transcripts [5].

Reassociation kinetics was used early on to study and compare global properties of tissue transcriptomes [1],[6]. From those studies it was concluded that ∼20,000 mRNAs are expressed in each cell or tissue, and that roughly 90% of all mRNAs are common between two tissues, drawing the first conclusions on tissue transcriptome compositions [7]. Later studies of tissue transcriptomes using SAGE [8] identified ∼1,000 ubiquitously expressed genes (i.e. expressed in all cell types examined) and concluded that tissue-specific transcripts make up roughly 1% of mRNA mass of cells. Focusing on colorectal cancer cell lines, for which the deepest coverage was available, it was estimated that half of all mRNA transcripts in these cells came from the 623 most highly expressed genes. Comparing mRNA expression levels across panels of human and mouse tissues by microarrays, Su and coworkers identified tissue-specific genes for each tissue, and estimated that ∼6% of genes were ubiquitously expressed, and that individual tissues express 30–40% of all genes [9]. Using additional microarray data, expression of ∼8,000 genes was detected in each tissue but as few as 1–3% of these were detected in all tissues [10]. Similar conclusions were drawn from a second mouse tissue atlas [11] that identified ∼1,800 genes as ubiquitously expressed. Altogether, microarrays and SAGE have been quite successful in identifying tissue and cell specific genes [8]–[12]. However, the discrepancy between estimates of the composition and characteristics of tissue transcriptomes obtained by microarray and SAGE methods on the one hand and reassociation kinetics studies on the other has not been explained.

Deep sequencing of RNAs (RNA-Seq) has recently been used to quantify gene and alternative isoform expression levels [13]–[17]. In RNA-Seq, all RNAs of a sample (or, more often, polyA^+^ RNAs) are randomly fragmented, reverse transcribed, ligated to adapters and then these fragments are sequenced. Gene expression levels can then be estimated from the number of sequence reads deriving from each gene [15]. Expression estimates from RNA-Seq are quantitative over five orders of magnitude and replicates of mouse tissues are highly reproducible [13]. Compared to microarrays, RNA-Seq is more sensitive, both in terms of detection of lowly expressed and differentially expressed genes [15],[18], and expression values from RNA-Seq correlate better with protein levels [19]. The greater accuracy and coverage of the expressed transcriptome makes this method suitable for addressing global features of transcriptomes.

We recently studied alternative isoform expressions across tissues using RNA-Seq and found both a very high frequency of alternative splicing and extensive tissue regulation of the expression of alternative mRNA isoforms [14]. Here we instead focused on a gene-centric analysis of transcript composition and complexity. The highly quantitative nature of RNA-Seq has motivated us to revisit the longstanding questions regarding the composition of tissue transcriptomes, as well as the expression of long non-coding RNAs, the variability in 3′UTR length, and the association between these features and gene function.

## Results

### Excluding 3′ UTR reads yields more accurate gene expression estimates

We investigated the transcriptomes of a diverse collection of human and mouse tissues and five breast and breast cancer cell lines that were recently sequenced at a depth of roughly 20 million short reads per sample using RNA-Seq protocols (Table S1). Gene expression was initially estimated by calculating read density as ‘reads per kilobase of exon model per million mapped reads’ (RPKM) [13]. These estimates are typically performed using common gene annotations (e.g., RefSeq) with the entire annotated transcript representing the ‘exon model’. These expression level estimates may however be confounded by the expression of shorter isoforms due to alternative cleavage and polyadenylation (Figure S1A and S1B). We found that excluding annotated 3′UTRs – which will sometimes vary between mRNA isoforms as a result of alternative cleavage and polyadenylation – enabled estimation of expression levels that correspond more closely with quantitative RT-PCR measurements (Figure S1C). We noted that removing the 3′UTR from calculation of gene expression yields a >2-fold change for over one thousand genes (Figure S1D), and that the effect of 3′UTRs on expression estimates does not seem to be a technical issue caused by secondary structure in the 3′UTR (Figure S2). We therefore advocate excluding UTRs from such estimates, and all subsequent gene expression estimates described here excluded 3′UTR regions.

### Ubiquitous expression of ∼8,000 human genes

**Figure 1:**
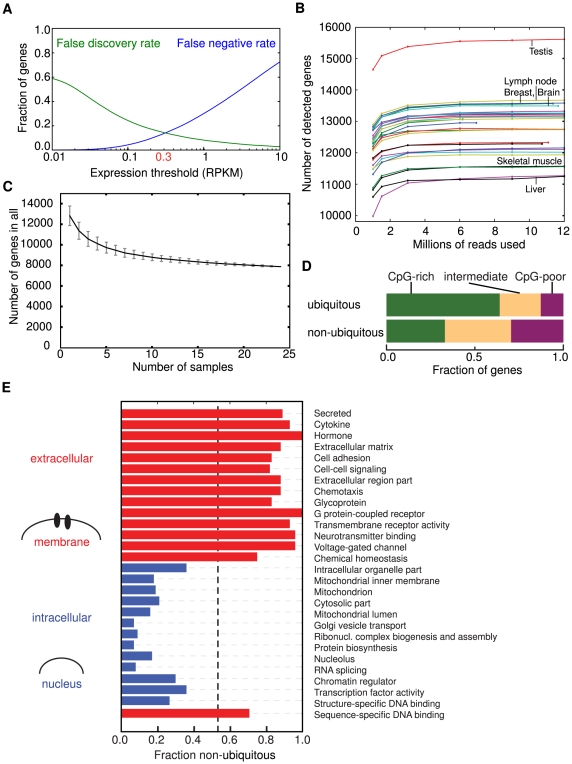
Functions of ubiquitous genes. (A) False discovery and negative rate for the detection of genes as a function of detection threshold used, demonstrating how a threshold of 0.3 RPKM was chosen. (B) The number of genes detected (>0.3 RPKM) at different sequencing depths. Each curve represents a sample. Above 3 million reads the sequence depth matters little for how many genes are detected as expressed. (C) The number of ubiquitous genes (expressed >0.3 RPKM in all samples) as a function of the number of samples used. Error bars show the standard variation, black line the mean. (D) The fraction of genes among ubiquitous and other genes with CpG-poor (purple), intermediate (yellow) or CpG-rich (green) promoters. (E) Illustration of subcellular localizations aligned to protein functional and localization categories for significant categories enriched in ubiquitously expressed genes (blue) and genes that were only expressed in one or a few tissues (red). For each category we have plotted the fraction of all genes that were not ubiquitous (the overall fraction of non-ubiquitous genes are shown as a vertical dashed line). Extracellular functions and membrane functions were highly enriched for non-ubiquitous genes while intracellular functions were dominated by ubiquitous genes. The categories shown are a subset of all significant categories listed in Dataset S2 and S3.

We next sought to answer how many genes are expressed in a tissue or cell type. A comparison between the expression levels of exons and intergenic regions was used to first find a threshold for detectable expression above background (Figure 1A, algorithm in Figure S3), yielding a threshold RPKM value of 0.3 which balances the numbers of false positives and false negatives. For individual samples, we obtained threshold values between 0.2 and 0.8. As it is difficult to identify untranscribed DNA regions with confidence [20],[21], it is very possible that the background was overestimated. Applying the threshold 0.3 RPKM, the number of genes expressed in most human and mouse tissues varied from 11,000 to 13,000, corresponding to roughly 60–70% of RefSeq protein-coding genes (Table 1). These gene number estimates were stable across different sequencing depths (Figure 1B) and therefore represent bona fide tissue differences. Testis was a clear outlier, expressing more than 15,000 different genes (84% of RefSeq genes). As many as 7,897 genes (42%) were observed to be expressed in all tissues and cell lines (Dataset S1). The corresponding number for Ensembl annotation was 8,214, or 38% of protein-coding genes (Ensembl is an automated gene annotation system, whereas RefSeq is manually curated). Each ubiquitous gene was typically expressed at roughly the same order of magnitude in all tissues, suggesting that there were few problems with genes being considered ubiquitous when they were really specific to one or a few tissues but had a leaky, non-functional expression elsewhere (Figure S4). While we observed small numbers of reads for 8 genes known to have leaky transcription [22],[23] in several tissues, these genes were all too weakly or narrowly transcribed outside their main tissue to be detected as ubiquitous. The estimated number of ubiquitously expressed genes appeared to plateau as the number of samples used was increased to the full set of 24 (Figure 1C). The detection threshold used affects the number of genes detected (Table 1), and the number of detected ubiquitous genes can vary by up to ∼2,000 genes depending on threshold used. The number of samples is large enough that background is unlikely to cause relatively tissue-specific genes to be detected in every sample. These differences between thresholds therefore most likely reflect the presence of low-abundance RNA species. The number of ubiquitous genes we detected is much greater than the ∼1,000 shared genes identified by SAGE [8] and the 1–6% of genes from microarrays [9]–[11], but is in relatively good agreement with the ∼10,000 shared genes estimated by reassociation kinetics [6] and the 3,140 to 6,909 estimated from ESTs [24] (the higher number came from a cutoff of presence in 16 out of 18 tissues, used to remedy uneven EST sequencing across tissues). The increased number of ubiquitously expressed genes compared to SAGE and microarrays most likely results from the increased depth of mRNA-Seq data and improved detection of lowly expressed genes [22]. The number of genes expressed in a tissue ranged from 11,199 to 15,518 genes (Table 2), so a majority of the genes expressed in a specific tissue or cell type are ubiquitously expressed genes. These genes contribute ∼75% of the polyA^+^ RNA molecules in most tissues (Table 3), although this fraction was higher in the cancer cell lines, perhaps as a result of their elevated metabolic rate.

**Table 1:** Number of expressed and ubiquitous genes for various minimum expression thresholds.

Threshold RPKM	In all 24 samples	On average per sample
0.01	10,233	14,885
0.1	9,205	14,011
0.2	8,466	13,327
0.3	7,897	12,859
0.4	7,388	12,489
0.5	6,946	12,170
0.6	6,535	11,887
0.7	6,176	11,633
0.8	5,898	11,401
0.9	5,618	11,189
1	5,361	10,989
2	3,510	9,432
3	2,513	8,340
4	1,931	7,493
5	1,548	6,804

**Table 2:** Number of human genes expressed per tissue.

Tissue/Cell	Number of genes*	Fraction of genes*	Ensembl genes†
Skeletal muscle^1^	11,276	0.61	11,953
Liver^1^^,^ 3	11,392	0.61	12,191
BT474^4^	11,844	0.64	12,808
MB435^4^	11,847	0.64	12,726
HME^5^	12,084	0.65	12,920
T47D^4^	12,205	0.66	12,983
Heart	12,209	0.66	13,159
MCF7^4^	12,281	0.66	13,216
Adipose tissue	12,553	0.68	13,503
Colon	13,016	0.70	14,052
Cerebellum^2^^,^ 3	13,132	0.70	14,043
Kidney	13,235	0.71	14,177
Brain^1^	13,298	0.71	14,107
Breast	13,406	0.72	14,537
Lymph node	13,534	0.73	14,686
Testes	15,518	0.84	16,869

*annotations from RefSeq, protein-coding genes.

†number of protein-coding genes, annotations from Ensembl.

1 number of genes detected in mouse: skeletal muscle 11,799; liver 11,201; brain 13,626.

2 standard deviation for samples from different individuals: 106.

3 mean number for different individuals.

4 breast cancer cell line.

5 human mammary epithelial cell line.

**Table 3:** Fraction of mRNA pool by copy number from ubiquitous human genes.

Tissue/Cell	Fraction ubiquitous
Liver^2^	0.31
Heart	0.66
Brain	0.74
HME^4^	0.75
Breast	0.75
Skeletal muscle	0.76
Cerebellum^1^^,^ 2	0.76
Testes	0.77
Kidney	0.78
Adipose tissue	0.81
Colon	0.82
Lymph node	0.84
T47D^3^	0.87
MB435^3^	0.89
MCF7^3^	0.89
BT474^3^	0.90

1 standard deviation for samples from different individuals: 0.01.

2 mean number for different individuals.

3 breast cancer cell line.

4 human mammary epithelial cell line.

### Functions of ubiquitous and non-ubiquitous genes

To characterize the set of ubiquitously expressed genes we had identified, we looked for functional enrichment compared to genes expressed only in a subset of the tissues analyzed (hereafter called non-ubiquitous). The protein products of human ubiquitously expressed genes were more likely to have intracellular localization and to be involved in metabolism and other core cellular functions such as macromolecule synthesis, general transcription and vesicles (Figure 1E). Genes that were expressed in only one or a few tissues were more often secreted or membrane-bound (Figure 1E; Dataset S2 and S3), suggesting that cellular contacts and communication are mediated more often by specialized tissue-specific components. Interestingly, an exception to this inside-outside rule was sequence-specific DNA binding proteins, which are nuclear yet seldom ubiquitously expressed. Among these transcription factors we found that POU, homeobox and forkhead genes had the fewest ubiquitously expressed members, consistent with roles in specifying cell and tissue identity [25], whereas e.g. basic-leucine zipper factors were more often ubiquitous (Table 4). Functional characterization of housekeeping genes has been done in the past [26],[27] (and indirectly by [28]), with comparable results, although transporters were found to be relatively tissue-specific in one study [26]. Rather than looking at ubiquitous expression, that study compared the mean number of tissues where the genes were expressed, which could explain the difference. Ubiquitous genes often had CpG islands near their promoters (Figure 1D), as has been observed previously for ubiquitous and developmental genes [29]. The set of ubiquitous genes with CpG-poor promoters were not enriched for any GO category compared to all ubiquitous genes, nor were those with CpG-rich promoters. These observations suggest that ubiquitous expression is a better indicator of housekeeping functions than promoter CpG content. Together, these analyses suggest that much of the internal cytoplasmic machinery and most nuclear functions are common to most or all tissues, and that a large portion of the differences between tissues lie primarily in expression of receptors and ligands that mediate communication, and in a subset of sequence-specific DNA binding transcription factors.

**Table 4:** Expression of sequence-specific transcription factors.

Transcription factor classification	Number of genes	Fraction non-ubiquitous
POU	14	0.93
Homedomain	239	0.89
Forkhead	41	0.78
ETS	28	0.71
Helix-loop-helix	86	0.67
p53 family	42	0.67
Other	152	0.66
Nuclear hormone receptor	47	0.66
Zinc finger, C2H2	623	0.61
High mobility group	39	0.59
IPT/TIG^1^	17	0.47
Basic-leucine zipper	53	0.42

1 IPT: Immunoglobin-like fold shared by Plexins and Transcription factors; TIG: Transcription factor ImmunoGlobin.

### Estimating the fraction of the transcriptome devoted to specific functions

**Figure 2:**
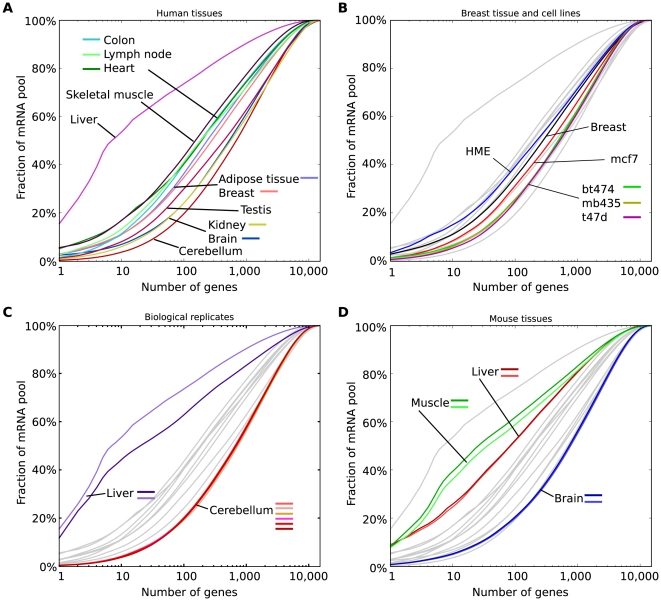
Complexity of tissue transcriptomes. (A) The fraction of all mRNAs derived from the most highly expressed genes for a number of mouse and human tissues. For example, the 10 most expressed genes in mouse liver contribute 25% of all mRNAs in that tissue. (B) Same as A, but with cell lines from breast. HME is a transformed cell line from normal mammary epithelium, breast is the normal tissue, the others are breast cancer cell lines from invasive ductal carcinoma. Gray lines are the tissues in A. (C) Same as B, but with 2 human livers and 6 human cerebellar samples from different individuals, to illustrate the degree of reproducibility in this type of plot and little inter-individual variation. (D) Same as B, but with three tissues from mouse.

As RNA-Seq expression measurements are highly quantitative, we also explored tissue transcriptome composition in terms of mRNA abundance classes [1] and the extent to which mRNA populations are dominated by a few highly expressed genes. Genes were sorted according to their expression and the fraction of the total cellular polyA^+^ RNA pool devoted to the most highly expressed genes was determined. This analysis showed that mRNA expression in both tissues (Figure 2A) and cell lines (Figure 2B) followed a continuous distribution rather than separating into distinct abundance classes as reported in previous studies (e.g. [1],[6]).

In muscle and liver transcriptomes, a small number of genes contributed a large fraction of the total mRNA pool, e.g. the ten most highly expressed genes in liver and muscle made up roughly 20–40% of the mRNA population. Other tissue transcriptomes were more complex, with the ten most highly expressed genes contributing only 5–10% of the mRNAs in brain, kidney and testis. The remaining tissues had intermediate levels of complexity (Figure 2A). The breast cancer cell lines had similar or greater complexity than normal breast tissue (Figure 2B). Biological replicates in both human and mouse tended to have highly similar complexity distributions (Figure 2C, 2D). Mouse tissues had somewhat similar profiles to corresponding human tissues (Figure 2D), although a much higher expression of several acute-phase genes in both human liver samples shifted their curves toward lower complexity compared to mouse liver. We conclude that kidney, testes and brain tissues have more complex transcriptomes due to the expression of more genes and with less dominance of a few highly expressed genes, whereas liver and muscle tissues are the least complex and express fewer genes, with more dramatic contributions of highly expressed genes.

**Figure 3:**
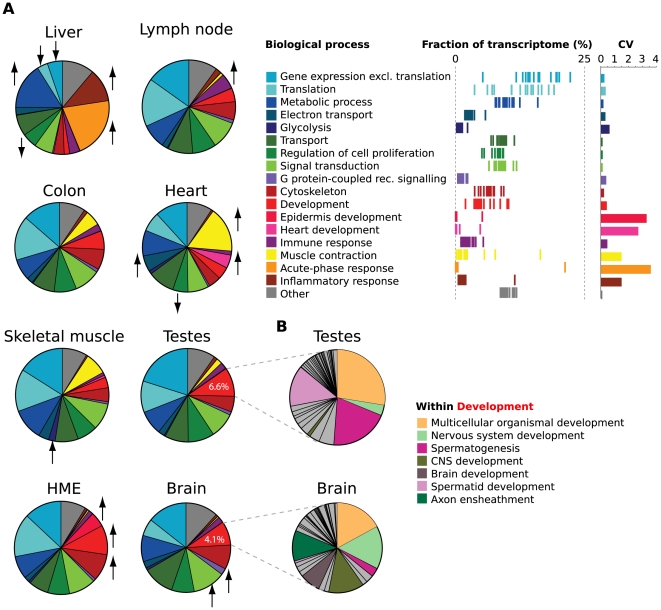
FRACT analysis of tissue transcriptomes. (A) Pie graphs show estimated fraction of cellular transcripts deriving from genes belonging to a set of top-level Gene Ontology Biological Process categories for 7 human tissues and 1 cell line. Fractions were estimated from read density (RPKM) of Ensembl transcripts for each gene. Names of categories, distribution of transcriptome fraction across the samples (each line is a sample), and the coefficients of variation are shown at right. Biological processes with significantly higher or lower densities in individual tissues and cell lines are denoted by arrows. (B) FRACT analysis of sub-categories of the top-level ‘Development’ category in brain and testes.

We next asked what fractions of total cellular mRNA are allocated to genes involved in different biological processes across the different tissues and cell lines. For this purpose, we developed a tool called FRACT (Functional Relative Allocation of Transcripts) that assesses relative gene expression from RNA-Seq read density for arbitrary sets of genes or broad gene ontology (GO) categories (results for a subset of tissues are shown in Figure 3A). This analysis provided a perspective on the functional priorities of cells in each tissue, since allocating a large fraction of the polyA^+^ RNA content in a cell (and likely of translational capacity) to one functional category represents a major investment of cellular resources. For some categories, including ‘metabolic process’, ‘transport’, and also ‘regulation of cell proliferation’, FRACT allocation varied relatively little across the tissues and cell lines (as measured by the coefficient of variation, CV, of the transcriptome fraction), consistent with the expected ‘housekeeping’ functions of these gene categories. Other categories had a far higher fraction of transcripts allocated to them in one tissue than in others, e.g. immune response (high in lymph node), muscle contraction, heart development and electron transport (all high in heart), and signal transduction and G protein-coupled receptor signaling (both high in brain). These examples, representing more specialized activities expected to be of increased importance in the corresponding tissues, provided a molecular-level validation of the integrity of the tissue samples and protocol used. In some cases, differences not readily apparent from the broad GO categorization shown in Figure 3A, could be detected by finer sub-classification of categories – an example is shown in Figure 3B.

**Figure 4:**
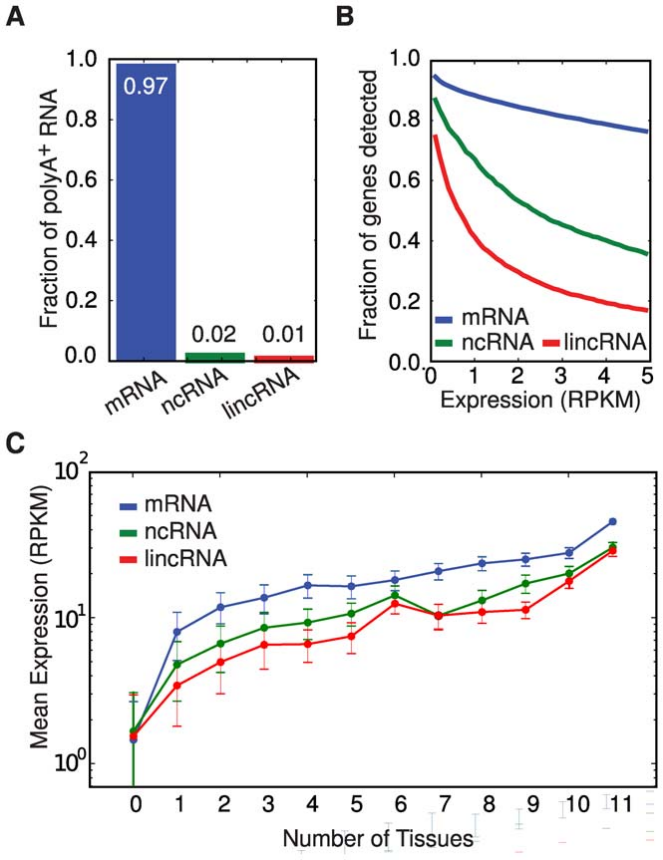
Non-coding RNA expression. (A) Relative fractions of polyA^+^ transcripts from protein-coding RNA (mRNA), curated non-coding RNA (ncRNA) and lincRNA, presented as the mean across human tissues. (B) The number of genes above a particular RPKM threshold (in one or more tissues) as a function of the threshold. (C) The maximum tissue expression level of mRNAs, curated ncRNAs and lincRNAs as a function of the number of tissues with detected expression. The average and standard deviations of the max expression levels in each group of genes are shown.

We also investigated the expression of thousands of large non-coding RNAs (ncRNAs). These genes were found to contribute a small fraction of transcripts to polyA^+^ transcriptomes compared to mRNAs (Figure 4A) as a result of their considerably lower expression levels (Figure 4B). These levels are lower than for mRNAs for all degrees of tissue-specificity (Figure 4C).

### Tissue-specific gene expression is fairly well conserved

Muscle and brain tissues from human and mouse were observed to have similar expression and FRACT distributions (Figure 2D and data not shown), raising the question of the extent of conservation of tissue-specific expression patterns. We compared global gene expression levels between human and mouse tissues and observed high correlations between expression of orthologous genes between human and mouse (Pearson correlation 0.76 for muscle, 0.77 for liver and brain). When different tissues were compared (e.g. human brain vs. mouse muscle) substantially weaker correlations were observed (Pearson correlations in the range 0.47 to 0.61). These observations indicate a fairly strong overall conservation of gene expression levels between mouse and man, consistent with previous studies based on microarrays [30].

### 3′ UTR length varies 3-fold between different functional groups of genes

**Figure 5:**
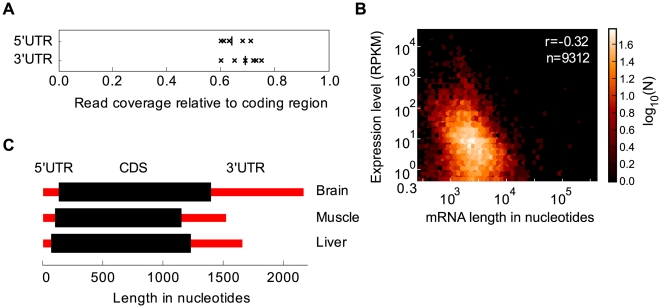
Variation in tissue transcriptome structures. (A) Read density in RefSeq gene annotation in the untranslated regions (UTRs) divided by that in the coding region (CDS) for the samples with least 3′ bias (mouse brain, muscle, embryonic stem cell and embryoid body; human adipose tissue and heart). Vertical lines indicate mean values. (B) Plot of mRNA length against abundance in mouse liver, showing that short mRNAs tend to have more copies. Pearson correlation and the number of mRNAs plotted are listed. (C) Expression-weighted average lengths of all mRNAs in three mouse tissues.

The lengths of mRNAs were studied by mapping the reads to coding and untranslated regions. Using RefSeq annotations, the density of reads in untranslated regions was lower than in coding regions (Figure 5A), suggesting that expression of mRNAs with UTRs shorter than or distinct from those annotated in RefSeq is common. We therefore estimated the lengths of the UTRs as their relative number of reads to coding regions using the annotated coding region length. Mouse data from [13] was chosen for this analysis as this dataset had little 3′ bias (Figure S5). In all three mouse tissues studied, significant negative correlations were observed between expression level and transcript length (−0.31 in liver and muscle, −0.16 in brain; all tissues p<10^−87^), showing that shorter mRNAs tend to be expressed at higher levels (Figure 5B). This result agrees with that from reassociation kinetics data [31]. Weighting each gene by the expression level to obtain length estimates for the bulk mRNA population in tissues to obtain the average mRNA length in each tissue, we found that brain mRNAs have longer 3′UTRs on average than liver and muscle mRNAs, by 300–400 nucleotides (Figure 5C).

**Figure 6:**
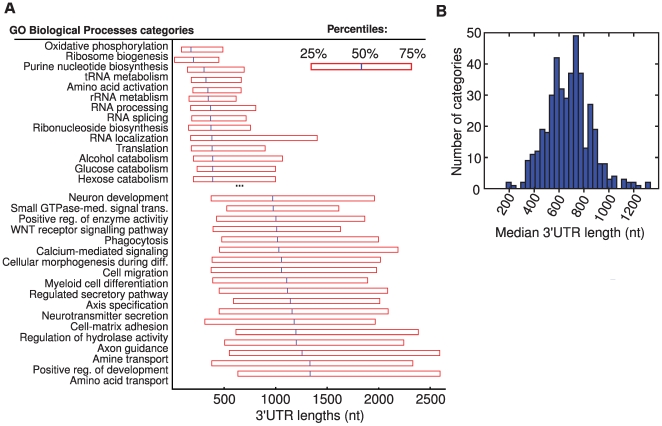
Associations between UTR lengths and protein functions. (A) The length distribution of 3′UTRs for genes in categories with the shortest respectively longest UTRs. The 25, 50 and 75% percentile lengths for each GO biological process category are presented. (B) The distribution of median lengths across all GO biological process categories.

To assess the protein functions encoded by transcripts with long or short UTRs, we calculated the median length of 5′ and 3′UTRs of genes associated with each GO biological process category (Figure 6B and data not shown). Transcripts coding for proteins involved in metabolism and RNA processing had the shortest UTRs (medians below 500 bp), while the longest median UTR lengths were observed in transcripts encoding proteins involved in development, morphogenesis and signal transduction (Figure 6A). The median lengths in the longest categories ranged between 1000 and 1500 nt, i.e. two- to three-fold longer than for typical metabolism- or RNA processing-associated transcripts. Some of these differences might reflect an increased role for 3′UTR sequences in localization of proteins to specific membrane locations, likely to be more common for proteins involved in signal transduction and morphogenesis than for metabolic or RNA processing-associated proteins, which are typically cytoplasmic or nuclear, respectively. These differences could also reflect differences in the complexity of translational regulation among these classes of genes.

## Discussion

A surprise in our analysis was the large number of ubiquitous genes found expressed in all tissues and cell lines, and that these genes account for a majority of the mRNA pool. This pattern suggests that tissue identity derives less from expression of distinct sets of genes in different tissues than was previously thought. Ubiquitous genes can still vary in relative expression levels between tissues however, and in expression of alternative mRNA isoforms [14]. Although a still limited set of tissue and cell lines was available for this meta-analysis (24 in total), the observation appears robust to inclusion of additional tissues (Figure 1C). Many genes had a low and rather constant expression across tissues. This could mean our expression detection was affected by subpopulations of cells, limiting the extent our conclusions can be extrapolated to single cells, but it could also indicate the existence of a large population of lowly but universally expressed genes. One subpopulation that could potentially impact these estimates would be organism-wide cell types. For example, blood-related cells may be found in all vascularized tissues and genes specific to these cells may be detected as ubiquitous. Our study limited this effect by requiring ubiquitous genes to also be detected in cell lines. Future analyses of pure cell populations could definitely assess the contributions of common cell types. When single-cell transcriptomes (like [32]) are available for multiple cell types, it will be possible to identify the core set of genes expressed in every mammalian cell. Still, our analyses of tissue transcriptomes points to a higher number of core genes even in individual cells than previously inferred.

Transcriptome complexity varied substantially across tissues, with brain, kidney and testis having higher complexity in that they expressed more genes and had more diverse mRNA populations. This increased transcriptome complexity may stem from the presence of more heterogeneous cell types in brain and testis or from a need for more diverse protein repertoires. The lower complexity observed in liver, muscle and heart presumably reflects more specialized functions of these tissues. Our FRACT analysis estimated the fraction of mRNA populations devoted to biological processes that are more specific for muscle and liver cells, such as muscle contraction, metabolism, electron transport and acute-phase response. At this point we have only static pictures of the functional allocation of mRNA resources across tissues and cell lines. Following the dynamic regulation of mRNA allocations during developmental or disease progression would therefore be of great interest, and might lead to robust gene expression signatures that are diagnostic of cellular state.

Many studies (e.g. [33],[34]) cite the existence of three distinct abundance classes of mRNAs, originally observed by reassociation kinetics [1],[6] (reviewed in [7]). Although we detected mRNA expression levels that varied across several orders of magnitude, we observed no separation of mRNAs into distinct expression level classes, instead finding a continuum of expression levels. Similarly, no separation into distinct expression classes was observed in SAGE data (Figure 4 in [35]), although the authors discussed the larger impact of sequencing errors. This discrepancy with reassociation kinetics analyses may result from the limited number of data points used in these earlier studies, in conjunction with line fitting algorithms that could artificially add inflection points [1],[36].

Previous studies using ESTs and microarrays have found a bias towards the usage of longer 3′UTRs in brain tissues [14],[37] and found that 3′UTR length can be dynamically regulated in response to activating and mitogenic signals [38]. The short read sequencing data allowed us to estimate the average lengths of transcripts in different tissues and we found that brain expressed mRNAs with 3′UTRs 300–400 bp longer on average than in other tissues. An important factor seems to be the brain-specific expression of genes with long 3′UTRs (data not shown). Perhaps this is required in a tissue where many mRNAs are transported far away from the nuclei, or the variety among neurons requires a large regulatory capacity housed in the UTRs. Interestingly, transcripts coding for specific protein functions seem to require longer 3′UTRs and 5′UTRs, including proteins involved in axon guidance which have on average almost three times the UTR length of ribosome biogenesis genes [39], suggesting extensive UTR-based regulation, e.g. of translation and/or mRNA localization, in this class of genes [40],[41].

It was striking how many protein-coding genes were expressed in all samples studied, even including many transcription factors. This pattern could help in identifying determinants of cell identity and responses, as ubiquitous genes are less interesting candidates and could be discarded or separated when clustering samples by gene expression. It could also make it easier to select candidate disease genes after genetic linkage or association studies as ubiquitous genes are less involved in hereditary diseases [42]. Furthermore, it accentuates the importance of cell communication as a regulatory mechanism, as these components are mostly restricted to particular tissues and cell types and play a role in ‘calculating’ what state a cell should have [43], information that is then transmitted through a relatively static interior of the cell. These components have relatively recent origins as a result of their importance in multicellular organisms [28],[44], and sit on the periphery of the protein interaction network, conveying information directly to and from the center consisting of highly connected and generally ubiquitously expressed genes [45]–[47].

## Methods

### Short-read RNA sequence data

We used short read data from human tissues from [14] (SRA002355.1) and [18], mouse tissues from [13] (downloaded from http://woldlab.caltech.edu/html/rnaseq), mouse embryonic cell and body data from [16] (http://grimmond.imb.uq.edu.au/mESEB.html) and cerebellum data from non-schizophrenic humans from [48]. See respective papers for details on library preparation, sequencing and general read mapping statistics. The data from [18] were mapped to build hg18 with bowtie [49] with setting –best and ambiguous reads were removed. Two human brain samples were used. The sample with lower sequencing depth from a mix of individuals was used in the comparison with RT-PCR data, while the deeper sample was used everywhere else.

### Gene expression estimates

We mapped read positions onto gene models and estimated gene densities as the number of reads divided by the number of read start positions. We used only reads that mapped uniquely to the genome, and only positions where a read could potentially map uniquely counted toward exon length. For testing different ways of measuring gene expression (by removing different parts of the gene structure), we selected a set of genes with >2 exons and only one annotated isoform in RefSeq whose expressions had been measured by the MicroArray Quality Control project [50] in the same two samples, UHR (universal human reference) RNA and brain. Cleavage and polyadenylation sites are from [51],[52]. RefSeq or Ensembl gene annotations without 3′UTRs were then used for all gene expression estimates. For genes with multiple splice variants, we fitted an RPKM value to each variant by least square regression and used the sum of the expression of all isoforms (Figure S6). Isoforms that did not overlap directly but were grouped only through overlap with a third isoform were not considered to represent the same gene. All Pearson correlations were calculated based on log-transformed expression. False discovery and false negative rates were estimated using the algorithm presented in Figure S3, which seeks to correct for the presence of spurious reads mapping to non-expressed genes. The extent of leaky ubiquitous transcription by comparison of the ubiquitous set of genes to shuffled controls (Figure S4).

### Gene ontology and CpG content

For three mouse tissues, we calculated RPKM values in the same way as had been done for the human ones. Mouse genes were matched to human orthologs using Entrez Gene. A list of acute-phase genes was taken from http://www.informatics.jax.org. DAVID [53] was used for finding enriched gene ontology categories. Categorization of promoters by CpG content was performed as described in [29]. Transcription factor annotations are from [54].

### Non-coding RNA

RefSeq gene annotation was used for protein-coding RNA (i.e. accessions starting with NM_) and curated non-coding RNA (NR_). We used the liftOver tool from the UCSC genome browser to obtain human positions for lincRNA regions from [21].

### Transcriptome analysis with FRACT

GO annotations for Ensembl transcripts were downloaded from Ensembl (BioMart). The read density for each transcript in each tissue was distributed among its annotated GO categories (total transcript density/no. GO categories for the transcript). GO categories were sorted by the total transcriptome density across tissues and cell lines, and the 400 categories with greatest density (accounting for 94% of total density) were aggregated into 17 broad classes; the remaining categories (6% of total transcriptome density) were aggregated into an “other” class (see Dataset S4 for mappings). The total density of transcripts devoted to each class in each tissue was tabulated. The coefficient of variation in the fraction of each transcriptome devoted to different classes was computed, and a Z-score for each class was computed to identify particular tissues which devote a significantly different fraction of the transcriptome to particular classes (|Z-score|>2).

### Length of the untranslated regions

The UTR lengths were calculated as the number of reads in a UTR divided by the number of reads in CDS multiplied by the CDS length. For the expression weighted average gene lengths, we used the CDS length from Refseq gene annotation, but weighted according to the expression of each gene. To see the correlation between mRNA length and abundance, we took the CDS length from RefSeq annotation for gene isoforms and added UTR length according to the distribution of reads in the three regions. Only those expressed above 0.3 RPKM were included, in order to exclude genes with few reads that could drive an artificial correlation. To compare 3′ bias between samples, i.e. to what extent genes get more reads as you go in the 3′ direction, we plotted the average read density for all genes (weighted so that each gene contributed equally) across the coding region and fit a line y = kx+m where y = read density, x = location along coding region, and k/m is a measure of 3′ bias.

## Supporting Information

Table S1 Tissue transcriptome data used
(0.25 MB PDF)

Figure S1 Gene expression estimates using different gene models
(0.66 MB PDF)

Figure S2 Folding of 3′UTR and expression level estimates
(0.19 MB PDF)

Figure S3 Estimation of false discovery and negative rates at different expression levels
(0.38 MB PDF)

Figure S4 Estimation of false discovery and negative rates at different expression levels
(0.24 MB PDF)

Figure S5 Read density across genes
(0.17 MB PDF)

Figure S6 Gene expression for genes with multiple mRNA isoforms
(0.19 MB PDF)

Dataset S1 Ubiquitously expressed human genes
(0.45 MB XLS)

Dataset S2 Enriched gene ontology categories among ubiquitous genes
(0.16 MB XLS)

Dataset S3 Enriched gene ontology categories among non-ubiquitous genes
(0.14 MB XLS)

Dataset S4 Functional Relative Allocation of Transcripts
(0.17 MB XLS)
