# Supplementary material for: An Abundance of Ubiquitously Expressed Genes Revealed by Tissue Transcriptome Sequence Data
Source: PLoS Comput Biol. 2009 Dec 11;5(12):e1000598. doi: 10.1371/journal.pcbi.1000598 (PMC2781110; doi:10.1371/journal.pcbi.1000598)
Supplement: Table S1 — Tissue transcriptome data used (0.25 MB PDF) [file pcbi.1000598.s001.pdf]

Supplemental Table 1. Tissue transcriptome data used

Sample	# reads that map to genome uniquely	Source	Figures															
			1A	1B	1C	2A	2B	2C	2D	3A	3B	4	5A	S1	S2	S3	S4	S5
Human brain, low coverage	4,399,037	Wang et al. 2008		X	X									X			X	
UHR	4,356,896	Wang et al. 2008		X	X									X	X		X	X
Human adipose tissue	18,404,196	Wang et al. 2008	X	X	X	X				X		X	X			X	X	
Human lymph node	16,515,702	Wang et al. 2008	X	X	X	X				X		X				X	X	
Human skeletal muscle	15,014,322	Wang et al. 2008	X	X	X	X				X		X				X	X	X
Human heart	11,828,357	Wang et al. 2008	X	X	X	X				X		X	X			X	X	
Human testes	19,232,158	Wang et al. 2008	X	X	X	X				X	X	X				X	X	
Human brain	11,282,682	Wang et al. 2008	X	X	X	X				X	X	X				X	X	X
Human colon	18,449,121	Wang et al. 2008	X	X	X	X				X		X				X	X	
Human liver	13,123,679	Wang et al. 2008	X	X	X	X		X		X		X				X	X	X
Human breast	11,020,683	Wang et al. 2008	X	X	X	X	X			X		X				X	X	
HME	13,168,691	Wang et al. 2008		X	X		X			X							X	
BT474	12,247,760	Wang et al. 2008		X	X		X			X							X	
MCF7	10,724,379	Wang et al. 2008		X	X		X			X							X	
MB435	13,123,679	Wang et al. 2008		X	X		X			X							X	
T47D	10,499,080	Wang et al. 2008		X	X		X			X							X	
Human liver	33,553,178	Marioni et al. 2008		X	X			X									X	
Human kidney	32,876,644	Marioni et al. 2008	X	X	X	X						X				X	X	
Human cerebellum	15,914,965	Mudge et al. 2008	X	X	X	X		X				X				X	X	
Human cerebellum	17,426,793	Mudge et al. 2008		X	X			X									X	
Human cerebellum	5,845,502	Mudge et al. 2008		X	X			X									X	
Human cerebellum	17,497,308	Mudge et al. 2008		X	X			X									X	
Human cerebellum	16,421,177	Mudge et al. 2008		X	X			X									X	
Human cerebellum	6,568,735	Mudge et al. 2008		X	X			X									X	
Mouse brain 1	14,488,583	Mortazavi et al. 2008		X					X				X					X
Mouse liver 1	13,133,047	Mortazavi et al. 2008		X					X				X					X
Mouse muscle 1	16,632,815	Mortazavi et al. 2008		X					X				X					X
Mouse brain 2	26,519,332	Mortazavi et al. 2008							X									
Mouse liver 2	17,673,013	Mortazavi et al. 2008							X									
Mouse muscle 2	16,632,815	Mortazavi et al. 2008							X									
Mouse embryonic stem cells	31,974,136	Cloonan et al. 2008											X					
Mouse embryoid bodies	29,180,840	Cloonan et al. 2008											X					

Abbreviations: UHR, universal human reference; HME, human mammary epithelium
