# Supplementary material for: An Abundance of Ubiquitously Expressed Genes Revealed by Tissue Transcriptome Sequence Data
Source: PLoS Comput Biol. 2009 Dec 11;5(12):e1000598. doi: 10.1371/journal.pcbi.1000598 (PMC2781110; doi:10.1371/journal.pcbi.1000598)
Supplement: Figure S1 — Gene expression estimates using different gene models (0.66 MB PDF) [file pcbi.1000598.s002.pdf]

A

B

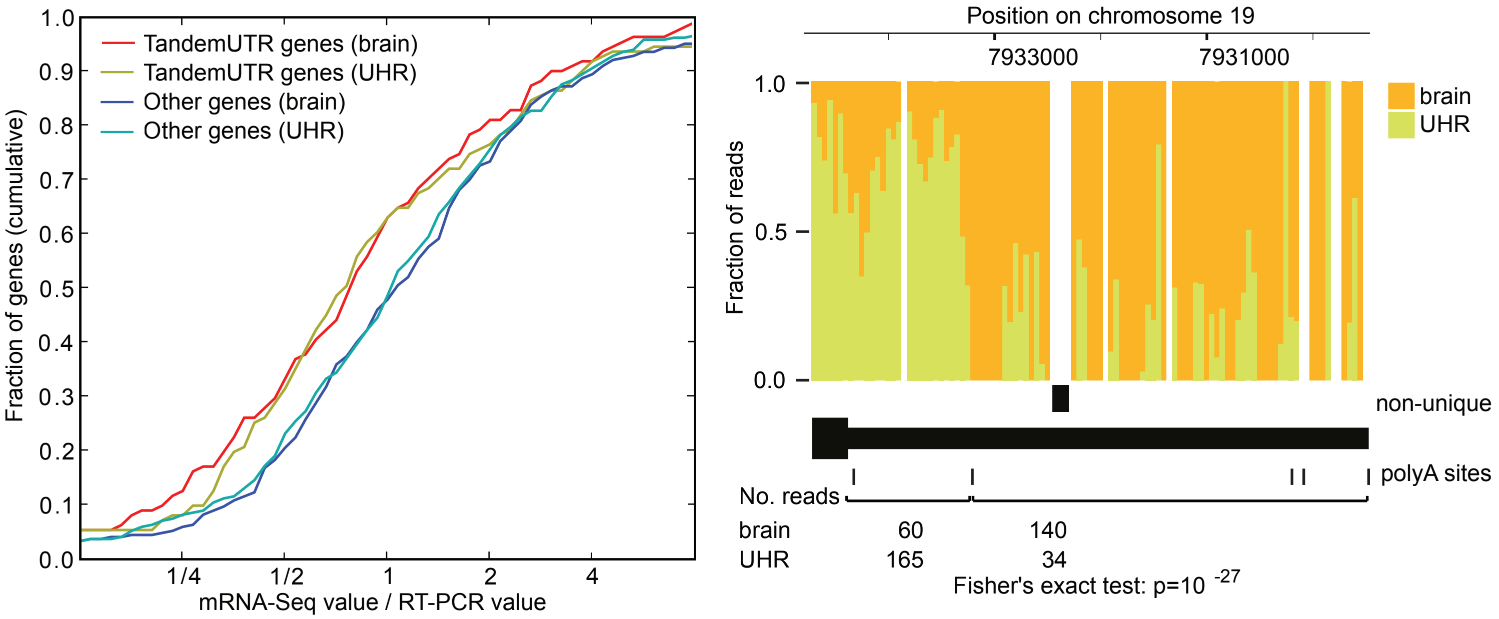

C

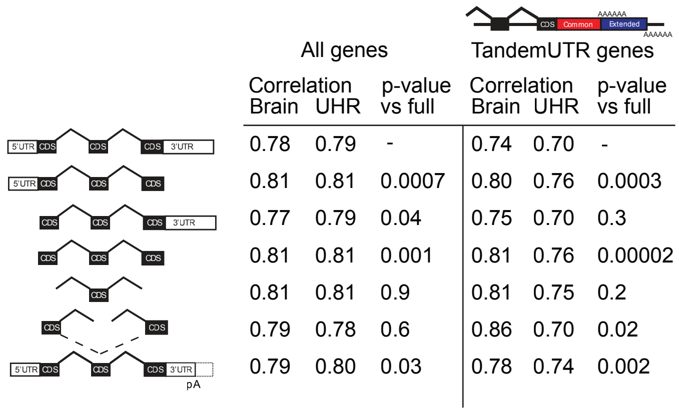

	All genes			TandemUTR genes		
	Correlation Brain	UHR	p-value vs full	Correlation Brain	UHR	p-value vs full
Full transcript	0.78	0.79	-	0.74	0.70	-
Without 3'UTR	0.81	0.81	0.0007	0.80	0.76	0.0003
Without 5'UTR	0.77	0.79	0.04	0.75	0.70	0.3
Coding region	0.81	0.81	0.001	0.81	0.76	0.00002
Internal exons	0.81	0.81	0.9	0.81	0.75	0.2
Constitutive exons	0.79	0.78	0.6	0.86	0.70	0.02
Truncated at first predicted poly(A) site	0.79	0.80	0.03	0.78	0.74	0.002

D

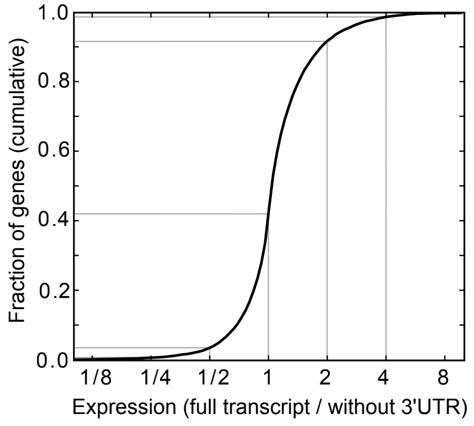

Supplemental Figure 1. Gene expression estimates using different gene models
(A) The expression of tandem UTR genes (multiple polyA sites in 3'UTR) is underestimated if the full transcript is used to calculate gene expression (p=0.02, *t*-test). (B) Alternative polyadenylation at the last exon of the gene *ELAVL1*, illustrating how a sample can express a shorter variant than the annotated one. (C) The effect on the gene expression estimated if part of the gene structure used in the calculation is removed. The p-value represents improvement relative to using the full transcript, when comparing with RT-PCR data and using the paired *t*-test. (D) The effect on perceived gene expression by removing the 3'UTR, for all genes.
