# Supplementary material for: An Abundance of Ubiquitously Expressed Genes Revealed by Tissue Transcriptome Sequence Data
Source: PLoS Comput Biol. 2009 Dec 11;5(12):e1000598. doi: 10.1371/journal.pcbi.1000598 (PMC2781110; doi:10.1371/journal.pcbi.1000598)
Supplement: Figure S2 — Folding of 3′UTR and expression level estimates (0.19 MB PDF) [file pcbi.1000598.s003.pdf]

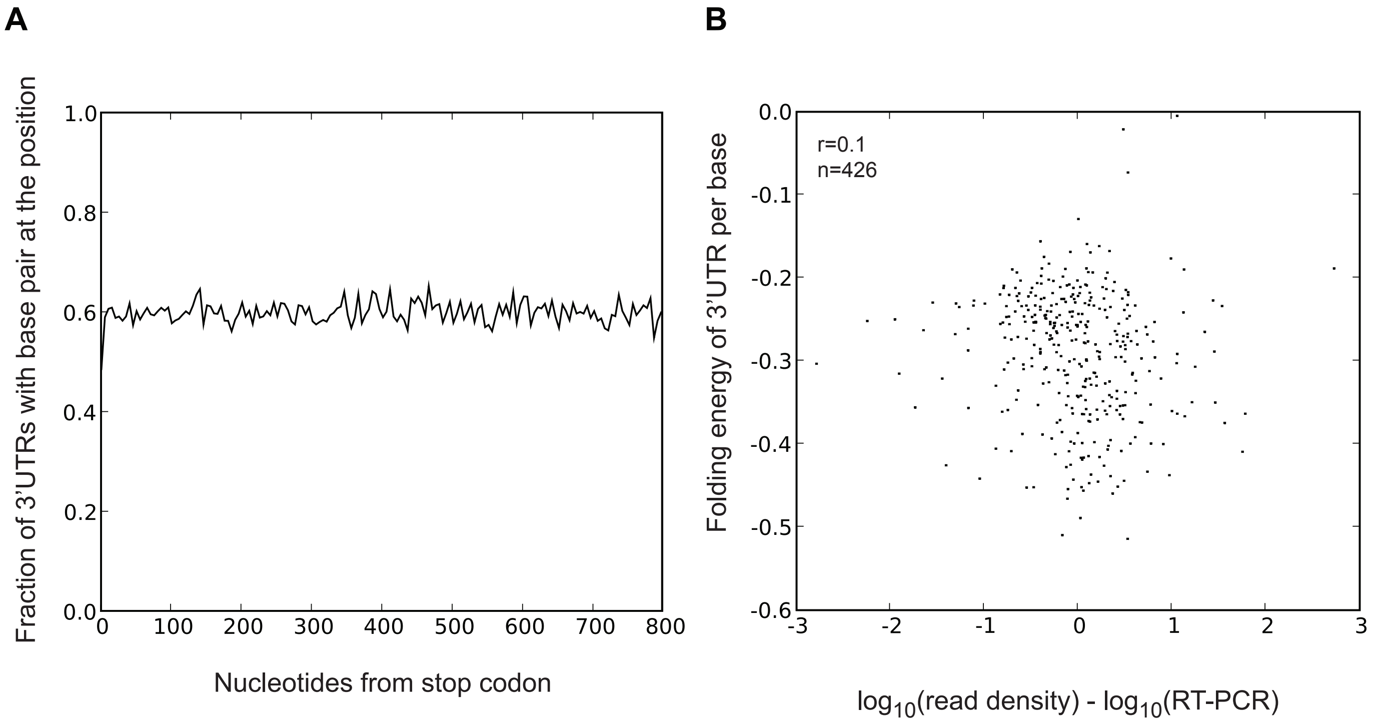

Supplemental Figure 2. Folding of 3'UTR and expression level estimates

(A) Base pairs predicted by RNAfold are evenly distributed in the 3'UTRs of 426 genes, unlike the read density distribution in the 3'UTR which is 50% higher near the stop (see Figure S3). The mean for each five nucleotides is shown. (B) The correlation is very poor between the stabilisation of secondary structure in the 3'UTR and the error read density in the 3'UTR. Data for UHR is shown. Stabilisation by secondary structure uses predictions by RNAfold (http://www.tbi.univie.ac.at/~ivo/RNA).
