# Supplementary material for: An Abundance of Ubiquitously Expressed Genes Revealed by Tissue Transcriptome Sequence Data
Source: PLoS Comput Biol. 2009 Dec 11;5(12):e1000598. doi: 10.1371/journal.pcbi.1000598 (PMC2781110; doi:10.1371/journal.pcbi.1000598)
Supplement: Figure S3 — Estimation of false discovery and negative rates at different expression levels (0.38 MB PDF) [file pcbi.1000598.s004.pdf]

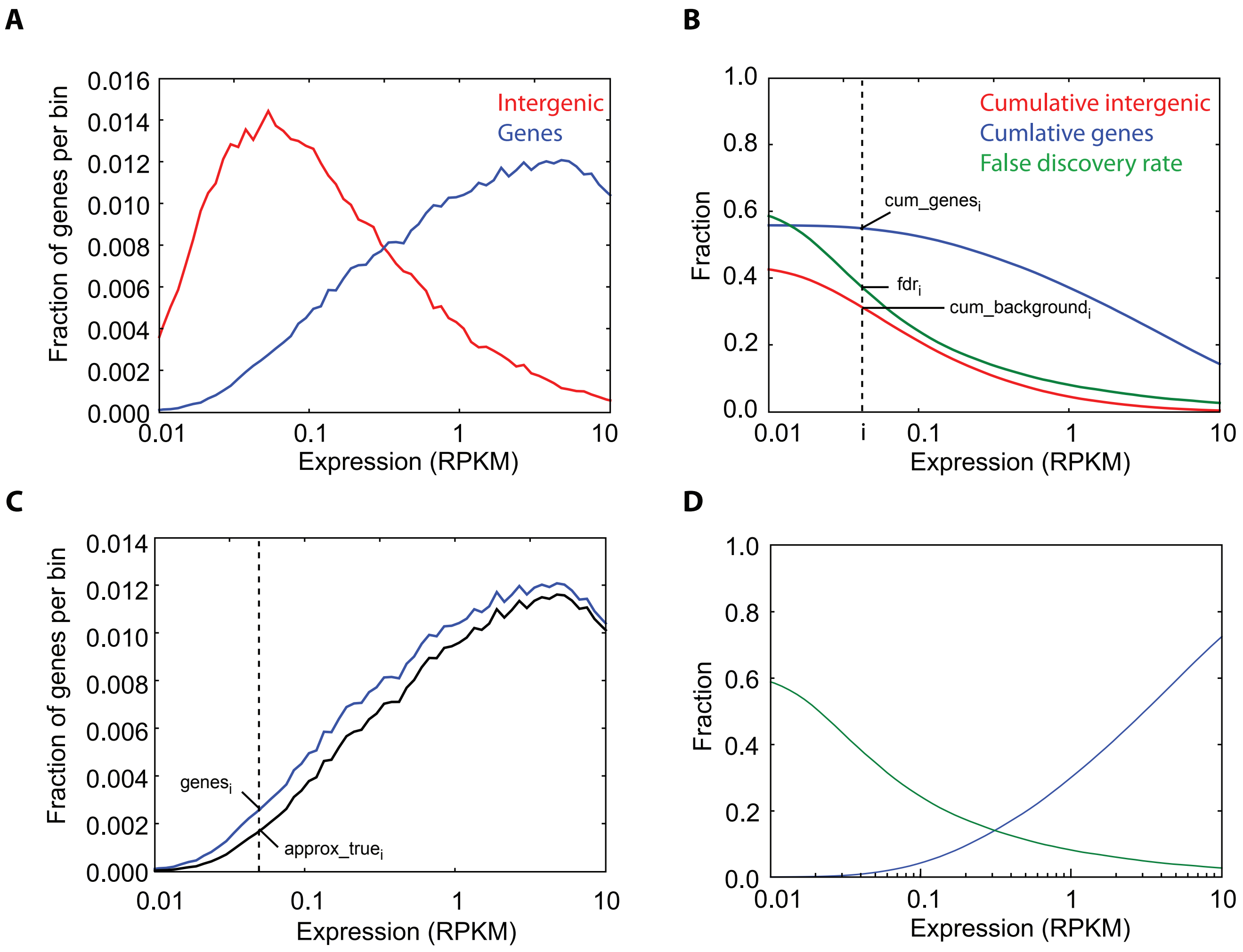

Supplemental Figure 3. Estimation of false discovery and negative rates at different expression levels

(A) Reads were mapped to Ensembl genes (blue) and intergenic background regions (red). The intergenic regions were matched to have the same length distribution and no ESTs mapping within regions. We binned the expressions of all genes and background regions across human tissues (for a list of tissues used see Table S1). We zoomed in on the effects at expression spanning between 0.01 to 10 RPKM for clarity. (B) Bins were converted to cumulative amounts of genes expressed above the expression levels for genes (cum_genes; blue) and controls (cum_background; red). A false discovery rate fdr (green) was calculated at each expression level, i, as:

$fdr_i = cum\_background_i / cum\_genes_i * (1-cum\_genes_i)/(1-cum\_background_i)$,

where the $(1-cum\_genes_i)/(1-cum\_background_i)$ factor corrects for the presence of negatives among the genes (i.e. the genes not expressed in the set of tissue studied). (C) The true number of expressed genes in each bin (black) was estimated from the observed numbers for Ensembl genes (blue, same as A) by multiplication of the latter by the false discovery rate. This estimate was converted to cumulative amount and the false negative rate was estimated as a function of expression level using the formula: false negative $rate_i$ = 1 - sum(from j=i; $approx\_true_j$) / sum(all j; $approx\_true_j$) . (D) The false discovery and negative rates, as shown in Figure 1A.
