# Supplementary material for: An Abundance of Ubiquitously Expressed Genes Revealed by Tissue Transcriptome Sequence Data
Source: PLoS Comput Biol. 2009 Dec 11;5(12):e1000598. doi: 10.1371/journal.pcbi.1000598 (PMC2781110; doi:10.1371/journal.pcbi.1000598)
Supplement: Figure S4 — Estimation of false discovery and negative rates at different expression levels (0.24 MB PDF) [file pcbi.1000598.s005.pdf]

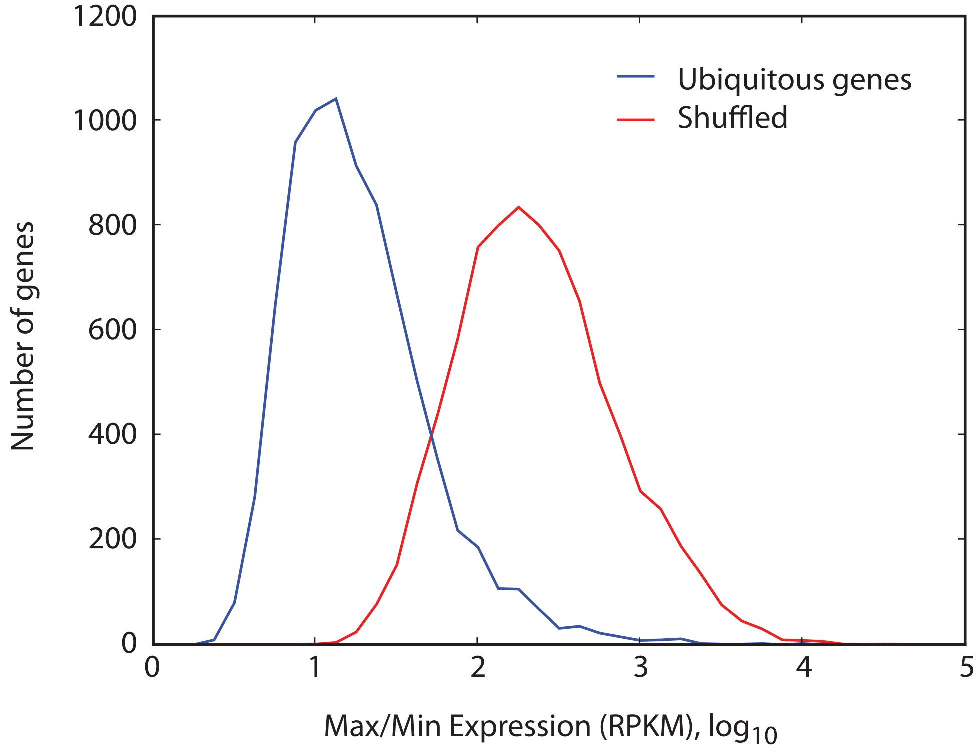

Supplemental Figure 4. Range of expression for ubiquitously expressed genes
The variation among different samples in gene expression of ubiquitously expressed genes. The blue curve represents the observed variation, which forms a single peak with few outliers, implying that this gene set contains few genes with a strong bias for a particular tissue. The red curve comes from shuffling the gene expression values for ubiquitously expressed genes for each sample, to indicate how the blue curve would have looked if each of the genes were not expressed at similar level in every sample.
