# Supplementary material for: An Abundance of Ubiquitously Expressed Genes Revealed by Tissue Transcriptome Sequence Data
Source: PLoS Comput Biol. 2009 Dec 11;5(12):e1000598. doi: 10.1371/journal.pcbi.1000598 (PMC2781110; doi:10.1371/journal.pcbi.1000598)
Supplement: Figure S5 — Read density across genes (0.17 MB PDF) [file pcbi.1000598.s006.pdf]

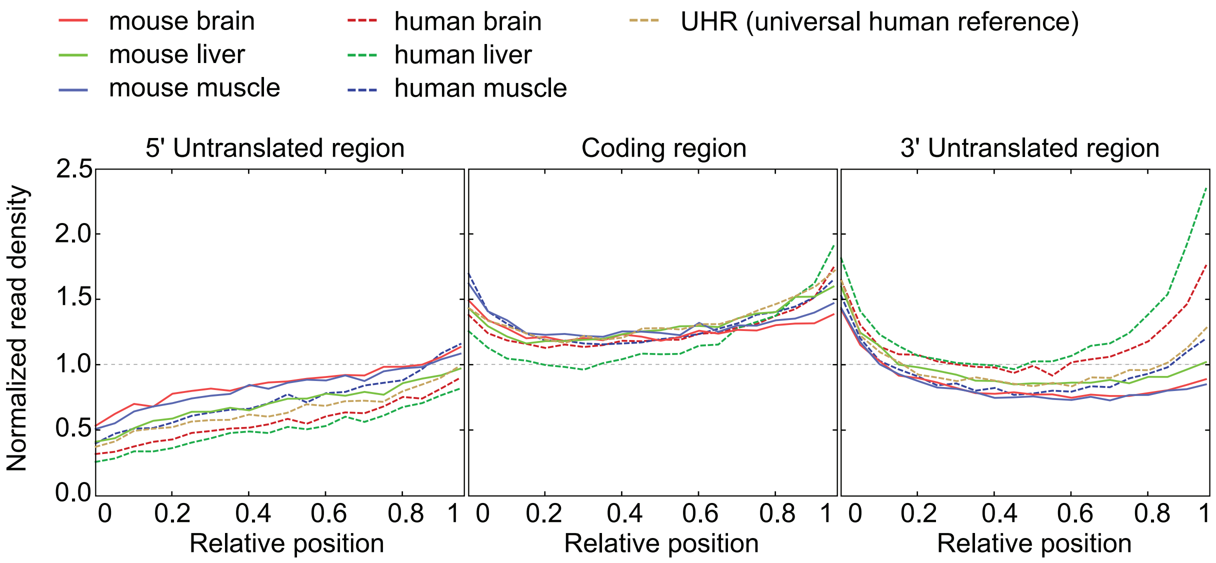

Supplemental Figure 5. Read density across genes

Distribution of read density across 5'UTR, coding region and 3'UTR, calculated for all genes weighted so that each gene contributes equally and normalized so that 1 corresponds to the average read density. We see less 3' bias in the mouse samples (solid lines) than in the human samples (dashed lines).
