# Supplementary material for: An Abundance of Ubiquitously Expressed Genes Revealed by Tissue Transcriptome Sequence Data
Source: PLoS Comput Biol. 2009 Dec 11;5(12):e1000598. doi: 10.1371/journal.pcbi.1000598 (PMC2781110; doi:10.1371/journal.pcbi.1000598)
Supplement: Figure S6 — Gene expression for genes with multiple mRNA isoforms (0.19 MB PDF) [file pcbi.1000598.s007.pdf]

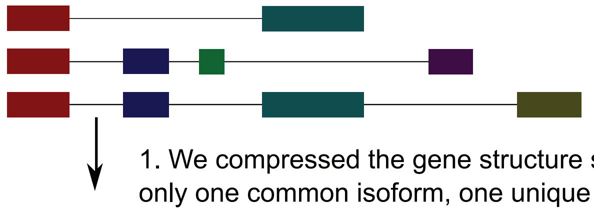

1. We compressed the gene structure so that there is only one common isoform, one unique to isoform 1 etc.

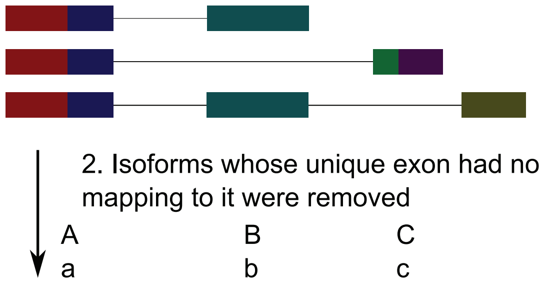

2. Isoforms whose unique exon had no read mapping to it were removed

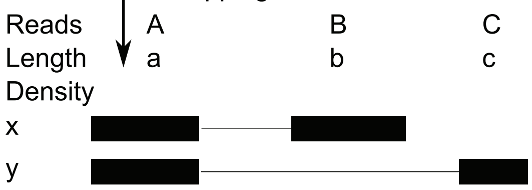

3. Regression: Minimized $(A - a(x+y))^2 + (B - bx)^2 + (C - cy)^2$ to get x and y

This was done by creating the matrices L, X and R where

$$L=\begin{pmatrix} a & a \\ b & 0 \\ 0 & c \end{pmatrix} \quad X=\begin{pmatrix} x \\ y \end{pmatrix} \quad R=\begin{pmatrix} A \\ B \\ C \end{pmatrix} \quad \text{and get } X=(L^T L)^{-1} L^T R$$

For cases where $(L^T L)^{-1}$ could not be solved, $(L^T L + I)^{-1}$ was solved instead, where I is the identity matrix

4. If x or y was negative, we set that density to zero and redid the regression for the remainig isoforms

5. We multiplied by 10^9/ total mappable reads to get a number in RPKM (reads per kilobase and million mappable sequences)

6. To get a number for the gene, we summed RPKM values for the isoforms

Supplemental Figure 6. Gene expression for genes with multiple mRNA isoforms

How gene expression from genes with multiple annotated isoforms was calculated. For simplicity, a case where only two isoforms are expressed is shown, but the algorithm was used also for cases with more isoforms.
